# A Consideration of the Casting Process, with Special Reference to Refractory Materials

**Published:** 1909-10-15

**Authors:** Marcus L. Ward

**Affiliations:** Ann Arbor, Mich.


### A CONSIDERATION OF THE CASTING PROCESS, WITH SPECIAL REFERENCE TO REFRACTORY MATERIALS.

BY MARCUS L. WARD, D.D.SC., ANN ARBOR, MICH.

Read before the New York Odontological Society, March 1909.

A little over two years ago Dr. William H. Taggart presented the cast gold inlay to the dental profession. Since that time probably 99 per ent. of the profession have, with undeveloped casting devices, rude investing compounds, and a circumscribed knowledge of the general principles involved in casting other materials, achieved enough success to justify them in ascribing the casting process to a most conspicuous place in intellectual concepts. The cast gold inlay has, even in the hands of the less successful, shown such possibilities that the advent of a refractory material which would meet the requirements of the process and a better knowledge of the principles involved is eagerly looked for.

During the two years that have elapsed, little of a scientific nature has been written on the process, while previous to the time of the introduction of the cast gold inlay the profession had little or no occasion to become familiar with the laws governing the casting of other materials.

Our knowledge of this new phase of dentistry must, then, be of comparatively recent origin and of a somewhat limited nature. About eleven months ago that never-tiring genius, Dr. Weston A. Price, presented the first of his series of articles containing the results of his experiments with the cast inlay. Two months later appeared the second, and five months later the third. In each of these articles appears so much of a scientific nature that to one who desires to pursue a different course of reasoning to a successful conclusion any one of them is positively dangerous. I do not hasten to undo his work, nor even question his result if all the details of his methods are carefully carried out; but in order that I may impress not only upon you, but through this channel upon the whole profession, that his methods are not the only ones pursued by engineers in such work, I must refer freely to his articles. He prefaces his first article by saying that—"To everyone with high ideals there certainly has frequently, if not continually, come a consciousness that gold and its alloys when built into large pieces for dental restorations, whether cast or fused, did not produce either exact or uniform restorations. This has been particularly serious and annoying in bridge work, and the extent of the error has been in proportion to the size of the piece, while the distress it gave the operator was in proportion to the height of his ideals. This constant error has been chiefly produced by the contraction of the metals on cooling and the contraction of the investment on heating, granting that the operator's technique has been faultless."

He goes on to describe how he has determined the amount of contraction there is on cooling metals from their melting point to ordinary temperature. After constructing an instrument which would magnify the change in dimensions, he cast several bars of gold and its alloys from wax models made on iridio-platinum pins which fitted tightly into the arms of his instrument. His first observation was that no two bars of gold were of the same dimensions, and often there was a large variation. He says, "This error proved to be due to two factors, chief of which was shrinkage of the investing material." To eliminate this error he constructed a form to cast into, which was made of fused pure quartz. He says of his work with this new form: "Bars of gold or its alloys cast in this fused quartz chamber, which itself had a relatively very constant dimension, showed in some cases close agreement to the total contraction as estimated from the readings of the expansions up from normal, but to my great disappointment different casts of the same metal showed different total contraction, which clearly indicated that another unsuspected source of error existed—for if conditions are the same, results must be the same. This difference in dimensions of the various casts of the same metal or alloy made in the chamber of fused quartz proved to be due entirely to the difference in *casting pressure*, and it was proved that any given pressure will produce constant results, other essential conditions remaining constant. For example, pure gold cast under one pressure contracted eighteen thousandths of an inch, and under another pressure only fourteen thousandths; under another very low pressure twenty thousandths. Eighteen-karat gold solder at a given high pressure contracted fifteen thousandths, and at a given low pressure eighteen thousandths on repeated casts. The great significance of this will be apparent to everyone, as well as its natural lesson, viz, that if we are to get constant results we must use constant pressure as well as have other conditions constant, and to secure the least contraction we must use as high pressure as possible without distortion of the investment—an important point, to be taken up later.'

Dr. Price has thus brought forcibly to the attention of the profession a number of things which to a great many may be new. First, he mentions that gold and its alloys contract on cooling. This should not be new to anyone, since a coefficient of expansion and contraction is worked out now for almost everything. Secondly, he mentions that investing materials shrink on heating, and that as a result of this shrinkage and the shrinkage of the gold on cooling no uniformity can be had in the dimensions of castings. Thirdly, he mentions that shrinkage of gold in cooling is decreased by an increase of pressure, and if a minimum amount of contraction is secured we must use as high pressure as possible without distorting the investment. He next calculates the actual pressure on an inlay of $\frac{1}{8}$ inch square, and finds that with an effective pressure of sixteen pounds per square inch only approximately $\frac{1}{8}$ of a pound is exerted on the inlay.

He then estimates that with a casting machine giving 11,000 lb. actual pressure on the gold there would be a total contraction on cooling of twenty thousandths per inch, while if it were capable of giving five and one-half pounds actual pressure there would still be a contraction of thirteen thousandths. He found none of our present investing materials suitable for such pressure nor capable of expanding enough to counteract the shrinkage of the gold on cooling. This grand procession over obstacles now culminates in the production of an artificial stone model which both expands sufficiently to counteract the shrinkage of the gold and enables one to use high pressure in casting.

We must agree that gold contracts on cooling, and that most investments shrink upon excessive heat, but to accept his method of correcting the evil would be to defeat not only the object of the designer of the process but some of the already accepted methods of engineers. The question which naturally arises in the mind of one who has been somewhat unsuccessful in this work is, "Must I reproduce the affected parts in artificial stone and cast directly upon the model to get perfect results?" To this let me say emphatically, No! We cannot conceal the fact that gold contracts on cooling, neither can we obscure our mortification at not finding investing materials that are uniform, if not of sufficient strength and free from change in volume. We still have these two phenomena to successfully combat if we are to keep the cast gold inlay in its present much-heralded position since most dentists meet with enough failures to keep them feeling that there is something wrong.

It is the opinion of the writer, however, that the amount of shrinkage which takes place in casting an ordinary sized filling is far less than Dr. Price's articles imply, and it is from this line of work that the profession is to receive its greatest benefits.* He has estimated that gold contracts about

*Dr. Price's measurements were taken upon a bar of gold, which is always more free to contract than a circular casting. It is upon this fact that the writer bases his opinion.—M. L. W.

twenty thousandths per inch, or 2 per cent. This appears somewhat high, though we will grant that it is correct for bars of gold. It has long since been observed by engineers that a great deal more allowance must be made for castings which are long in shape than those which are spherical, a wheel not being as free to contract as a long bar. It has also been observed that the contraction is in proportion to the amount of material used, a small casting having a much smaller coefficient of contraction than a large one. Sharp gives the table below to show the difference in coefficient of contraction between casting spur wheels of cast iron ten feet in diameter and those two feet in diameter:

CONTRACTION IN CASTING SPUR WHEELS IN CAST IRON.

| Extreme diameter of wheel casting. | Pitch in inches. | Width of teeth in inches. | CONTRACTION. | | | |
|---|---|---|---|---|---|---|
| | | | Total in inches. | Per foot of casting. | Per foot of pattern. | Per inch of casting. |
| ft. in. | | | | in. | in. | |
| 10 2⅜ | 3½ | 12 | 1.08 | 0.1059 | 0.1040 | 0.0088 |
| 6 2 9-16 | 3¼ | 9 | 0.54 | 0.0893 | 0.0886 | 0.0074 |
| 6 1⅜ | 3¼ | 11 | 0.375 | 0.0613 | 0.0610 | 0.0050 |
| 5 5 9-16 | 3½ | 11 | 0.345 | 0.0631 | 0.0628 | 0.0052 |
| 2 11⅞ | 3½ | 12 | 0.11 | 0.03896 | 0.03884 | 0.0032 |
| 2 4 15-16 | 3¼ | 9 | 0.115 | 0.0397 | 0.0396 | 0.0033 |

We observe from this that the wheel ten feet in diameter had a linear shrinkage of 1.08 in. per foot while the one two feet in diameter shrank only 0.115 of an inch per foot, a marked difference, as you see, not merely in the total amount of contraction but in the contraction per foot or inch.

If we now compute from this table the contraction of a wheel one-fourth inch in diameter, the size of a medium filling, we find it so infinitesimally small, and of such minor importance as compared to some other things in the casting process, that we might dismiss it from our minds while casting small fillings. For example, the contraction per inch of the wheel ten feet in diameter was 0.0088 in., and that of the small wheel 0.0033 in., the difference in coefficients of contraction between the large and small wheels being 0.0055 in. The difference in size of the two wheels is 93 inches. The difference in coefficients of contraction divided by the difference in size of the castings (0.0055÷93= 0.000059 in.) gives us an average reduction in coefficient of contraction, *with each inch reduction* in the size of the casting, of 0.000059 in.

Let us assume that every time we reduce the size of the casting one inch we reduce the coefficient of contraction (not the amount of contraction) 0.000059 in. If we begin now with the smallest casting given in the table (approximately 29 inches), and reduce it proportionately to the size of a medium-sized filling ($\frac{1}{4}$ inch) we find a coefficient of contraction per inch of 0.0016 in. [0.0033—(0.000059×28.75 in.)]. One-fourth of this amount will give us the actual contraction on a casting $\frac{1}{4}$ inch in diameter, which is 0.0004 inch, providing of course that our average reduction in coefficient of contraction is accurate. And why is it not reasonably accurate, since it is a principle which is verified by practical work in the foundry? The practical foundryman uses for large castings a pattern to construct his mold from which is about 0.10 of an inch per foot longer than he wants the casting to be. For castings of four inches or thereabouts he uses a pattern of the exact size he wants his casting, the small amount of shrinkage which takes place being cared for by the slight enlargement of the mold in removing his pattern. In castings considerable smaller than these he not only does not make his pattern larger, but makes it smaller by about 1-32 of an inch, the amount of shrinkage being so small that the rapping and removal of the pattern enlarges the mold more than is necessary to allow for the shrinkage of such small castings. Thus we see our deductions from the table of diminishing coefficients of contraction verified by practical work in the foundry.

We cannot say, then, that cast iron, brass, gunmetal, copper, lead, zinc, etc., that are used every day in the foundries, have fixed and definite coefficients of contraction. We must understand that with every reduction in the size of the casting there is a new and smaller coefficient of contraction. The natural question is, Why? Because the large casting remains hot longer than the small one. In other words, shrinkage is in proportion to the amount of material used, simply because the material for small castings chills the moment it strikes the mold.*

The lesson for us to draw from this work of the theoretical engineer and the practical engineer and the practical foundryman is quite forcible and obvious. We are to cast our gold into molds which have been heated sufficiently to eliminate mechanical moisture and allowed to cool down till they can just be handled with the hands without discomfort, if we are to place the casting of gold on a practical as well as scientific basis. From our computation of the shrinkage of a casting $\frac{1}{4}$ inch in diameter, we will assume that it shortens 0.0004 in. if made of cast iron. We will also assume that Dr. Price's determination of the shrinkage of pure gold is correct, 2 per cent., or twice that of cast iron. With this reasoning we can only calculate a shrinkage of 0.0008 in. if the casting were made of pure gold. Since the shrinkage of such materials as copper is quite well known, and known to be under that which we have accepted for gold, we have great reason to believe our calculations to be approximately correct. If, then, a filling $\frac{1}{4}$ inch in diameter shrinks 0.0008 in. and is seated in the center of the cavity, there will be 0.0004 of an inch to be filled with cement on each side. If the casting be a compound approximal one for a molar measuring $\frac{1}{2}$ inch, we can only calculate a total shortening mesio-distally of 0.0016 in. Imagine, if you will,

*Many small castings are of such a shape, and the sprue is of such a size that the linear shrinkage is greater than that of larger castings of other, shapes. This is because the smaller casting had its center chilled simultaneously with the periphery, and the result is that there is no hole in such a casting. The inlay, however small, does not belong to this sort of castings, its center being the last portion to cool, and the shrinkage can almost always be detected somewhere around the sprue.

such a cavity being prepared with all its walls inclining sufficiently to enable one to draw a wax impression, and a filling which had shortened from the extreme ends only 0.0016 of an inch. Do you doubt its going to place? It will do it so closely that the naked eye cannot detect it, if the cavity is properly prepared. These figures will not hold good for casts made in a red-hot mold, or if the gold is superheated. They hold good for casts made of gold heated above its melting point and a little below its boiling-point, and cast into a cool mold.

The writer has no desire to discountenance the use of artificial stone models and high pressures for cast work, but rather to impress you that it is not necessary to counteract the shrinkage of gold in cooling. There are a number of things which in the opinion of the writer influence the accuracy of a cast to a much greater extent than the shrinkage of the gold alone, though we must not forget that gold does shrink, and that it can be prevented beyond the limits of human error in other parts of the technique, if we will cast it into a dry but cool mold and carry out some of the details which will be taken up in order.

Experience has taught foundrymen that castings are much more free from defects if they use hot metal, although the crystalization is not so perfect in heavy castings. On the other hand, experience has taught them that the lower the temperature at which the fluid metal is poured into the mold, and the more rapidly is the mass cooled down to solidification, the closer will be the grain of the metal, the smaller the crystals, the fewer and less injurious the planes of weakness, and the greater the specific gravity of the casting. The writer has been able to demonstrate both of these experiences of the foundryman by casting gold heated with the electric arc and oxyhydrogen blowpipe and castin g it into fixed forms. We must judge then, from experience the lowest temperature at which gold can be carried into the mold so as to fill every cavity of it without risk of defect, if we are to meet the greatest number of the requirements of the process.

It is an *absolute necessity*, however, to have the gold as fluid as possible and to get the mold filled as quickly as it can be done, or the gold will solidify on its way to the remote parts of the mold. Next to the temperature of the mold, no three things are of greater importance than the temperature and the fluidity of the gold and the quickness of the operation. The writer's greatest objection to the present application of centrifugal force in casting, next to a varying pressure, is its slowness in exerting its pressure. If we are to follow an analogous science in this part of the work, we must have the gold as fluid as it will flow to remote parts of the mold, though not superheated, and carry it to place almost instantaneously. When melted with an ordinary blowpipe gold is not hot enough to be cast into a cold mold. Everyone should equip himself with an oxygen cylinder and yoke attachment for the blowpipe, if he is using an appliance not already equipped with one. It has been the writer's experience that gold should not be boiling when cast. With an oxyhydrogen blowpipe it is easy to boil gold. If the casting be a large one of irregular shape, and the mold has become cold instead of cool, then the gold should be cast upon the very first evidence of boiling, though in no case should gold be dancing up and down; it is not necessary in order to get the gold to its proper fluidity for even a cold mold, and will oftentimes prevent the gold being carried smoothly to place, besides adding to the shrinkage.

Any amount of heat will not make some golds fluid enough to cast perfectly. They appear sluggish even under the oxyhydrogen blowpipe. They are what the foundrymen would call "dull and slow-running metal." In nearly all the processes connected with metals and their alloys, constant reference is made not only to the temperature at which certain operations have to be performed to bring about certain results, but to the condition of the metal or alloy as regards its fluidity. It scarcely seems possible to exaggerate the importance of observing keenly the fluidity of the gold before attempting to make a casting of it. Gold to be suitable for casting purposes must have the property of being readily reduced to such a liquid state that it will permit of its being carried under pressure reasonable distances and still run into and occupy the most minute spaces in a mold. Gold in this condition can, other things being correct, be made into castings of the most intricate forms conceivable. If there are accumulations of a deteriorating kind about the gold, or if the gold has alloyed with something which makes it sluggish, fine margins and intricate forms cannot be produced. Gold must be free from accumulations on its surface, and it *must* be active under the blowpipe. The same is true of alloys of gold and platinum, a great many of which can be cast into certain shapes if they are just hot enough and of the proper fluidity.

The writer has made an effort to ascertain what impurities, if any, there were in the investments now in use which would contaminate a piece of pure gold while it was molten and being cast. A careful analysis was made of seven of the leading casting materials in use in the middle West. Each of them was found to contain silica and plaster of Paris as the principal ingredients, and all of them ferric oxide as an impurity. This was the only impurity that was found which could be considered as a contaminating substance. In one well-known material the ferric oxide was so evident that a number of casts were made to test its effect upon pure gold, and in each one the gold had changed in color, in fluidity, and melting-point. Other observations have been made upon investing materials in which the silica was chemically pure, and seldom has pure gold been capable of retaining its fluidity after being melted three times. Oftentimes golds having slightly different degrees of purity and fusibility have been mixed and cast, but there has not been uniformity in results. Sometimes they have combined properly with each other to form a clean fluid material, while at other times they have appeared to shrink unequally, and have not united into one homogeneous mass. These observations have led the writer to believe that dentists must keep at hand some practical means of purifying the gold regardless of the purity of the investing material or the perfection of their casting appliances. Gold and platinum and their alloys have their fluidity destroyed by repeated heating so as to make them too sluggish to make perfect castings, and if the silica has not been thoroughly washed with acids, the iron which is always present, and which unites with gold in all proportions, will furnish additional contamination. For this reason dentists must study the condition of the gold if they would make a success of casting. A number of different methods of purifying gold might be enumerated, but perhaps nothing will ever be of more service than about equal parts of potassium nitrate and borax. Both are clean white powders which can be mixed and kept in an accessible place.

If a casting is about to be made, and the gold is found to be too sluggish, the operation need not be delayed much over five minutes to purify the gold, while ten or fifteen minutes would raise the karat considerably. The process involves the oxidation of the base metals, the oxygen being furnished by the potassium nitrate, and these oxides being separated from the gold beneath the flux of borax. The gold should be melted and the mixture added a little at a time with one hand, and a blowpipe flame kept directed on the gold with the other, the gold all the time being kept melted. This should be continued in most cases for about five minutes, though it is often necessary to continue the process much longer. As soon as the last particles of nitrate and borax have been put on the gold the flame should be removed, to avoid its contaminating influence. Gold known to contain certain quantities of tin, silver, or lead could perhaps best be refined by the ammonium or mercuric chloride processes, but for iron, bismuth, antimony, zinc, etc., nothing excels potassium nitrate and borax.

Another thing of the most vital importance in the casting of gold into a mold as soft as we are at present obliged to use is the amount of pressure used and the accuracy with which it can be controlled. Dentists seemed to have grasped the idea that explosions of gunpowder, sudden generation of steam, crude applications of centrifugal force, the formation of small partial vacuums, compressed air made by swaging devices and the like are forces adapted to the casting of gold. It is true that some good work is done with many of these devices, but the person who expects to make a perfect cast every time he attempts it will learn sooner or later that he can only accomplish this with an appliance which exerts and maintains upon the gold a pressure which is definite. Gold cannot be carried into the mold with a low pressure one time and be thrown into it the next with much greater force any more than the engineer can allow molten metal to fall into the top of a sand mold one time and convey it to the bottom of the mold by vertical gates another time, and expect the same results from each method. A great deal might be said about this feature of the different appliances now furnished for casting gold, but the writer desires to refrain from it and to allow each one to draw his own conclusions.

The last phase of the subject which will be considered, and which is also of the greatest importance, is investing compounds and refractory materials. It can hardly be said that our knowledge of them is in a very satisfactory condition, or even that we know much about them beyond a few facts gathered through their use. We may observe that a given refractory material for engineering purposes is often called upon to fulfill conditions which are not only different, but exactly the reverse the one of the other. At one time it must resist an oxidizing, and immediately after withstand more or less of a reducing action. Sometimes it must resist the corrosive action of sulphuric acid, at others withstand the action of basic scoria, and at others, again, resist only the action of metals in fusing. An investing compound which meets the requirements of the casting process and bridge work should be strong, set readily, be free from shrinkage and expansion, and resist the action of the heat required to liquify gold and platinum and their alloys.

Though dental literature seems to be destitute of anything bearing directly upon the subject, an analysis of several products shows that silica and plaster of Paris have wisely been chosen as the two principal ingredients of our present investing compounds. In all plastic compounds there must be something which acts as a binder to the principal ingredients and their modifiers. In investing compounds we have evidence of this in the use of calcium sulfate plus one-half molecule of water, known as plaster of Paris, which in the presence of water takes up some of it and forms a crystalized variety. The combination of water with this salt to form the crystals sets the mass and acts as the binder for the silica. In our amalgams we have the binder formed by the union of mercury and silver and zinc, if the latter be present. In our phosphates the phosphoric acid and its modifications act with the oxide of zinc, while in many cases such as crucibles, muffle linings, etc., clay, a hydrous silicate of alumina, is used as a binder by making the clay anhydrous by baking. The question might be asked, Why is plaster of Paris not used alone? and the answer would be, Because it shrinks when heated. In addition to this it disintegrates when heated for any length of time, though we are forced to contend with this feature, and use the material with possible modifiers in small quantities as a binder for other materials.

In selecting other materials to use with plaster of Paris as refractories we naturally turn to the oxides, and of the oxides those of silicon, aluminum, calcium, and magnesium, and their compounds, are the ones with which we have to deal. Probably few realize the great quantities of rock and earthy materials that these four elements cover, silica, being the most abundant material of the earth except oxygen. The sands, quartzes, flints, opal, agate, and amethyst contain large quantities of silica. The corundum, emery, ruby, sapphire, etc., contain chiefly aluminum oxide. The feldspars are double compounds of aluminum and silica; mica is a double silicate of aluminum. The clays are chiefly silica and aluminum with ferric oxide. Chalk and whiting are calcium carbonate. Talc or soapstone is a compound of silica and magnesia. Asbestos is also a silicate of magnesia. Thus we see that almost all the materials which we would naturally turn to are compounds of silicon, aluminum, calcium, and magnesium.

It has been the experience of the metallurgical world, and nearly all are agreed, that it is hopeless to look for refractory materials which have given volumetric changes under heat, definite fusing-points, and absence of injurious impurities, among natural products. Such materials must be prepared artificially, since even small quantities of foreign substances often exert their influence to such an extent as to render given desirable products undesirable. One of the most striking examples we have of one material affecting another unfavorably is in the use of alumina, which alone is highly infusible, and silica, which alone is almost infusible. If 1 per cent. to 3 per cent. of alumina be mixed with silica the mass becomes fusible, while if we raise the amount of alumina to 40 per cent. or more, the mass again becomes highly infusible. If we review the effects which different elements constituting refractory materials have with each other, as well as with a number of other materials which may be used as modifiers, we find that the same element often produces exactly contrary effects according to the proportion in which it is present, and that there is nothing anomalous in those effects being produced. Alumina, when combined with iron or many other bases alone, makes infusible aluminates, but if silica be present it becomes more or less fusible. Almost the same phenomenon appears in the use of different sizes and shapes of the particles of silica, when silica is used alone, as we shall see a little later. This is but one of the many examples which might be given to impress the makers of investing compounds that they cannot rely upon natural market products and meet the requirements of the casting process, unless they watch them with chemical analysis, a good set of sieves, a microscope and micrometer.

The metallurgist who is accustomed to the use of clay for furnaces, muffles, etc., watches closely for a clay of a known composition and as to the mechanical arrangement of the particles. He knows that the plasticity of the clay is due to the fineness of the particles, the presence of alumina, and to water of combination. He also knows that for two materials having exactly the same chemical composition, one being coarse and the other fine, the coarse may be practically infusible while the fine may be more or less easily fuslble. He is also aware that the more porous the same substance is, the more infusible it will be. He knows, too, that the plasticity is destroyed by the presence of certain substances, such as magnesia, lime, and iron, and the refractory nature of it by sodium, potassium, lime, and magnesia. The makers of investing materials should profit by the workers in allied sciences, and get down to exactness in composition, exactness in the size of the particles, and exactness in the shape of the particles. One who is skilled in the plastics would turn to these phases of the problem at once, though they do not seem to form a part of dental knowledge at present.

Of the four substances mentioned as refractories, *silica* is the one most useful. It is very refractory, is of a desirable color, expands when heated, and is easily prepared, though it has one objection, that of having no binding quality. *Alumina* is very refractory, easily prepared, desirable to handle, though it shrinks when heated, and affects silica unfavorably as a refractory up to 40 per cent. *Magnesia* is one of the most infusible of substances, is easily enough prepared, and desirable to handle, but it shrinks markedly when heated. *Lime* is not used much in commercial operations, because the carbonate, the only form in which we have it, becomes caustic under heat, and when left to itself absorbs water and falls to powder. It is infusible, but is easily acted upon by silica, and affects plaster of Paris unfavorably. Silica is the only one of the four substances which we can rely upon to expand when heated, which practically means that it is the only rock or rocky material which will invariably expand under heat. Silica stands almost alone as a compound capable of being expanded under heat. Magnesia shrinks so much when heated that it should be excluded. Alumina also shrinks when heated, and, as we should naturally expect, its compounds do so too.

The writer has examined a number of the leading investing materials somewhat critically and with a view of presenting some suggestions to the makers of the same that would be of benefit. Nearly all of them are using silica and plaster of Paris, and yet no two of them appear alike. The reasons for this are obvious, since one can buy an indefinite number of grades of silica in the open market. A number of these investing compounds contain these two ingredients in proportions which appear quite right, though the problem which has confronted the writer before in the study of plastics seems to be unsettled by the makers of these investing compounds. The profession has given little attention to the physical phenomena in plastics, apparently thinking the chemical aspects the only ones needing attention, but the plan which the writer will suggest for making all investing materials will certainly give a good example of physical phenomena which need to be watched more closely. The products which we have now are mostly fine ones; a few have some coarse sand in them. An examination of the fine ones for streng th shows that after being heated they will scarcely hold together while being placed in a dynamometer. Rarely will a cylindrical piece of them one inch by one inch resist enough pressure to get the hands started on the dial of the dynamometer. An examination of the coarse ones shows them to be stronger though less uniform in their change in volume.

After separating the plaster of Paris from a number of these compounds and examining the shape and size of the particles of silica with the microscope, it was found that in most of the fine ones there was uniformity in the size and shape, while some of the coarse ones appeared like a conclomerate mass of coarse and fine particles without regard to the size or shape. After making a great many castings at different pressures, it was found that the castings made at the low pressures were the truest to shape, the higher pressures apparently causing the finer investments to be bulged in the manner indicated by the cross section of the tooth and filling exhibited this afternoon. It became apparent that even with the Taggart appliance, which has the pressure under absolute control, there was danger at times of distorting the investment unless the pressure was very low. Compounds of silica and plaster were prepared in which the percentage of silica was higher, and the result was a still weaker compound, though less free from shrinkage. The percentage of plaster was then increased, and the mass was stronger, but it shrank in proportion to the plaster used, modified more or less by the shape and size and arrangement of the particles of silica. The problem then appeared to be analogous to the preparation of concrete for pavement, if we were to get the maximum strength and get the mass so tightly filled in that the expansion of the silica would counteract the shrinkage of the plaster during heating. To carry out this plan, nine different grades of flint were obtained from Herman Behr & Co., 721 Beekman St., this city, and examined carefully with a microscope to see if there was uniformity in the sizes, and all the grades—Nos. FF, 240, 200, 000, 00, 0, ½, 1, 2, and 2½—appeared good, though all the particles were not of exactly the same size. An analysis was made to find if any injurious substances were present, and it was found that the average composition of the flint was about 97.2 per cent silica and 2.8 per cent. ferric acid, the most of which was removed by washing with hydrochloric acid. Three finer products than No. FF were purchased from W. H. Whittaker, 245 Front St., this city, bearing the trade names Nos. 19, 159, and 1900. Four other products were obtained from the Bridgeport Wood Finishing Co., of New Milford, Conn., and 72 Lake St., Chicago, bearing the trade names Nos. 3 | 100, 3DP, and 5 DP silex, as well as a very fine grade known as No. XXX lithowhite, all of which were almost pure silica, the only other substance being a small quantity of ferric oxid. Three important natural products and seven precipitated, chemically pure products were added to make an assortment of sizes. The No. 200 flint was selected as the coarser material to be used, and supposed to correspond to the gravel in concrete. All the finer products were examined microscopically to find a material which would correspond in size to the sand in concrete, and the No. XXX lithowhite was selected as the one to use. The different plasters were also examined microscopically to select one which would correspond to the cement in concrete, and French's impression plaster was selected for the trial.

When engineers determine the proportion of gravel, sand, and cement used in concrete, they select a gravel which is of a size adapted to their needs, and measure out a given amount of it. To this gravel they add water till it just fills the voids and appears at the top of the measured quantity. The water thus used represents the spaces between the particles of gravel, and also represents the minimum amount of sand which they are to use to fill these spaces. A given quantity of sand which will "fill in" well the spaces between the particles of gravel is now measured out, and the water poured into it, till all the voids are filled. The water thus used represents the amount of spaces in the sand, and the minimum amount of cement to be used to fill in these spaces.

Thirty cubic centimeters of the No. 200 flint were measured out, and water dropped into it from a burette, till it just appeared at the top. The amount of water used was 15 cc., which represented the amount of spaces in cubic centimeters. As is common with dry powders, the No. XXX silica was found to settle after being stirred with water to about one-half its original volume, so that to get 15 cc. of it wet, 30 cc. of the dry powder had to be used. To this amount 10 per cent. was added, as is customary with engineers to be sure that there is a slight excess of the second or filling material, making in all 36 cc. of the No. XXX silica. To 36 cc. of the No. XXX silica, water was then dropped from a burette till it just appeared at the top. The amount used was 16 cc., which represented the spaces in 36 cc. of the silica, and with a little added, represented the amount of plaster of Paris to be used as a binder.

The trial formula thus determined for the proposed dental concrete, then, was 30 cc. of No. 200 flint, 36 cc. of No. XXX silica, and 17 cc. of plaster of Paris. These ingredients were mixed thoroughly in the proportions named and the mixture was tried out. While it needed a little more of the No. XXX silica to make a smooth casting, the mixture showed the great advantage of using two carefully selected known sizes of silica in known quantities in preference to one size of silica or two unknown sizes. The same plan has been carried out with coarser flint to get an investment for bridge work with equally gratifying results, the result being a mass which was free from movement, more porous, and much stronger. If the plan is carried out carefully, the movement appears to be practically nothing, the expansion of the silica counteracting the shrinkage of the plaster. Other sizes of silica than those mentioned may prove to be better adapted to this work, but it will take considerable practical work to determine this.

Another class of compounds which may possibly be introduced into this work, but probably upon the same plan, is the different silicates of magnesium in the forms of asbestos, soapstones, etc. They are nice smooth compounds to handle, and with the ordinary heat appear to remain quite neutral in their movement, part of which appears to be due to the blocking up of the mass through their fibrous structure, and part to the arrangement of the silica and magnesia in such proportions as to render the shrinkage of the one neutral by the expansion of the other. There are a number of substances which can be used as modifiers in small quantities with plaster of Paris, though the virtue of most of them seems questionable.

An investing material made on the plan suggested seems to meet the requirements of the process when made of other sizes of silica, and with the co-operation of the makers of these compounds we should have some good ones in the near future, for both inlays and bridge work, which are free from movement, sufficiently strong, and more porous. If the makers of investing materials carry this plan out to a nicety they should, besides selecting carefully known sizes of silica or other material, wash the silica with acid to remove the iron, which is certain to be present, and other substances contaminating to gold which may be present in small quantities. This, however is scarcely necessary, if the flint and No. XXX silica continue to be mined in as nice a quantity as they are now. The flint has considerable iron in it, but it is completely covered up by the No. XXX silica, which is a most excellent product. A great many times the washing with acid will change the behavior of a product a great deal, so that the makers of these compounds must watch this phase of it with some care.

In conclusion, let me ask the manufacturers to try out this plan, using as a trial formula, by weight, Nos. 200 or 000, or 00 flint 10 parts, No. XXX silica 14 parts, model plaster 7 parts, and watch closely the amount of moisture in these ingredients before weighing.

And of the profession let me ask that each one purchase a small postal scale and a glass graduate, so that the water may be measured and the powder weighed in a moment's time, in such proportions as will make the investing material as thick as can be poured conveniently after having been mixed thoroughly.—*Cosmos.*

